# Predicting Breast Cancer Relapse from Histopathological Images with Ensemble Machine Learning Models

**DOI:** 10.3390/curroncol31110486

**Published:** 2024-10-24

**Authors:** Ghanashyam Sahoo, Ajit Kumar Nayak, Pradyumna Kumar Tripathy, Amrutanshu Panigrahi, Abhilash Pati, Bibhuprasad Sahu, Chandrakanta Mahanty, Saurav Mallik

**Affiliations:** 1 Department of Computer Science and Engineering, Siksha ‘O’ Anusandhan (Deemed to Be University), Bhubaneswar 751030, India; ghanarvind@gmail.com (G.S.); or amrutanshupanigrahi@soa.ac.in (A.P.); 2 Department of Computer Science and Information Technology, Siksha ‘O’ Anusandhan (Deemed to Be University), Bhubaneswar 751030, India; ajitnayak@soa.ac.in; 3 Department of Computer Science and Engineering, Silicon University, Bhubaneswar 751024, India; pradyumnatripathy@gmail.com; 4 Department of Information Technology, Vardhaman College of Engineering (Autonomous), Hyderabad 501218, India; prasadnikhil176@gmail.com; 5 Department of Computer Science and Engineering, GITAM Deemed to Be University, Visakhapatnam 530045, India; 6 Department of Environmental Health, Harvard T.H. Chan School of Public Health, Boston, MA 02115, USA; sauravmtech2@gmail.com; 7 Department of Pharmacology & Toxicology, University of Arizona, Tucson, AZ 85721, USA

**Keywords:** breast cancer relapse, cancer diagnosis, histopathological images, machine learning, ensemble learning

## Abstract

Relapse and metastasis occur in 30–40% of breast cancer patients, even after targeted treatments like trastuzumab for HER2-positive breast cancer. Accurate individual prognosis is essential for determining appropriate adjuvant treatment and early intervention. This study aims to enhance relapse and metastasis prediction using an innovative framework with machine learning (ML) and ensemble learning (EL) techniques. The developed framework is analyzed using The Cancer Genome Atlas (TCGA) data, which has 123 HER2-positive breast cancer patients. Our two-stage experimental approach first applied six basic ML models (support vector machine, logistic regression, decision tree, random forest, adaptive boosting, and extreme gradient boosting) and then ensembled these models using weighted averaging, soft voting, and hard voting techniques. The weighted averaging ensemble approach achieved enhanced performances of 88.46% accuracy, 89.74% precision, 94.59% sensitivity, 73.33% specificity, 92.11% F-Value, 71.07% Mathew’s correlation coefficient, and an AUC of 0.903. This framework enables the accurate prediction of relapse and metastasis in HER2-positive breast cancer patients using H&E images and clinical data, thereby assisting in better treatment decision-making.

## 1. Introduction

Globally, breast cancer is still among the most common malignancies affecting women. Among them, HER2-positive breast cancer stands out as a subtype defined by the overexpression of the HER2 protein. When cancer cells overexpress the HER2 protein, it can lead to HER2-positive breast cancer, a subtype of the disease that can grow more aggressively in tumors. Using histological photographs stained with H&E, one may physically inspect the tissue samples and aid in identifying and classifying the cancer cells [1,2]. Information like this might be useful for researchers in various ways, such as monitoring the progression of an illness, discovering novel therapeutic approaches, and evaluating the effectiveness of current therapies. Even with advancements in treatment methods, a major challenge in clinical practice is predicting the likelihood of sickness return [3,4]. Pathologists may learn a lot about the cellular architecture and structure of breast tumors from images captured after staining tissue samples with hematoxylin and eosin (H&E). Researchers have sought to develop deep learning (DL) and machine learning (ML) models for predicting breast cancer relapse using the mountain of data available in databases such as The Cancer Genome Atlas (TCGA). The most recent worldwide statistics from 2020 show that, surpassing even lung cancer, breast cancer has the highest incidence rate. Although targeted therapy has greatly enhanced the survival rates of breast cancer patients, around 30–40% of patients still face the challenge of relapse, and 10–15% ultimately succumb to cancer metastasis or relapse [5]. Timely identification of the reappearance and spread of cancer increases the effectiveness of treatment and substantially reduces the death rate. Due to the fast progress of AI and the increasing volume of multi-modal medical data, there is now a chance to detect and predict the relapse and spread of cancer at an early stage [6]. The prediction models should be further optimized to improve the discrimination ability and benefit, and prospective external validation studies should also be carried out before they are applied to clinical practice. Researchers conducted a comprehensive study using a network pharmacology-based strategy to predict therapy targets of Ecliptae Herba in treating breast cancer [7,8].

### 1.1. Existing Works

Sakri et al. [9] used the Breast Cancer Wisconsin Prognostic Breast Cancer (WPBC) Datasets for breast cancer relapse prediction. They employed ML techniques such as the Reduced Error Pruning Tree (REPTree), Naïve Bayes (NB), and K-Nearest Neighbour (KNN)-IBK in conjunction with particle swarm optimization (PSO). The results showed an accuracy rate of 81.3%, a precision of 88.3%, a recall of 93.4%, an F-score of 87.7%, and an area under the curve (AUC) of 0.820. Alom et al. [10] used the Breast Cancer Dataset to achieve a classification rate of 100% accuracy, 100% sensitivity, 100% specificity, and 1.00 AUC in breast cancer classification from histopathological images using an inception recurrent residual convolutional neural network (IRRCNN) that took into account DL techniques such as the Inception Network (Inception-v4), Residual Network (ResNet), and Recurrent Convolutional Neural Network (RCNN). Hong et al. [11] utilized a panel of eight microRNAs to predict the return of triple-negative breast cancer. They used ML techniques, specifically Logistic Regression (LR) and Gaussian mixture analyses, on the TCGA_TNBC, GEOD-40525, GSE40049, and GSE19783 datasets. The results showed an AUC of 0.89 for GSE40049 and 0.90 for GSE19783. Yan et al. [12] developed a prediction model in a primary cohort consisting of 498 patients with invasive breast cancer, and the data were gathered from July 2016 to September 2018 to predict pathological complete response and tumor shrinkage size in breast cancer considering an ML approach, i.e., LR, on Breast Cancer Cinoma (BCC) using the Harbin Medical University Cancer Hospital (HMUCH) dataset and resulted in a nomogram for HER2-positive and triple-negative breast cancer (TNBC) with an AUC of 0.820 and 0.785, respectively. Mosayebi et al. [13] proposed a new model for the prediction of relapse of breast cancer considering ML approaches, i.e., RF, Learning Vector Quantization (LVQ), NB, C5.0 DT, Multilayer Perceptron (MLP), Kernel Principal Component Analysis-SVM (KPCA-SVM), and SVM using Ministry of Health and Medical Education and the Iran Cancer Research Centre (MHME-ICRC) datasets. They resulted in 81.9% accuracy, 86.9% sensitivity, 77.7% specificity, 82.2% for the geometric mean of sensitivity and specificity, 76.9% PPV, 87.4% NPV, 82.0% for the geometric mean of PPV and NPV, 81.6% for the F-Value, and 77.4% AUC. Comes et al. [14] utilized DL approaches, specifically a Convolutional Neural Network (CNN) and Support Vector Machine (SVM), on DCE-MRI exams from the public datasets I-SPY1 TRIAL and BREAST-MRI-NACT-Pilot. The results showed an accuracy rate of 85.2%, a sensitivity rate of 84.6%, and an AUC of 0.83. Sanyal et al. [15] developed a weakly supervised temporal model that could predict distant breast cancer relapses using DL techniques (e.g., LSTM and XGBoost) on datasets curated manually and by natural language processing experts. The model achieved an AUC of 0.94, a sensitivity of 89.0%, and a specificity of 84.0%. The HEROHE challenge, organized by Conde-Sousa et al. [16] as a side event of the 16th European Congress on Digital Pathology, attempted to determine the HER2 status in breast cancer using solely H&E-stained tissue samples. The results showed 79% precision, 100% recall, a 79% F-Value, and 0.88 AUC. Multimodal prediction of five-year breast cancer relapse in women who receive neoadjuvant chemotherapy was proposed by Rabinovici-Cohen et al. [17]. Using a real-world retrospective dataset, the authors achieved 57% specificity, a 56% F-Value, 72% balanced accuracy, 41% PPV, 96% NPV, 93% sensitivity, and 0.75 AUC. The study also considered the DL approach known as CNN. Liu et al. [18] explored the use of color and texture features in histopathological images to predict the likelihood of breast cancer recurrence and metastasis. They used ML techniques such as RF, LR, and XGBoost on both a clinical dataset and formalin-fixed, paraffin-embedded (FFPE) samples from the TCGA. The results showed that the clinical dataset achieved 80% accuracy, 40% precision, 75% recall, a 50% F-Value, and 0.75 AUC. In contrast, the TCGA dataset achieved 73% accuracy, 33% precision, 60% recall, 42% F-Value, and 0.72 AUC. Yang et al. [19] suggested using DL to predict the risk of metastasis and relapse for HER2-positive breast cancer using CNN and ResNet50 on the datasets of the Cancer of the Chinese Academy of Medical Sciences (CAMS) and TCGA, respectively, using histopathological images and clinical data, with AUCs of 0.76 and 0.72. Lu et al. [20] presented a new model called Slide-Graph + that uses graph neural networks (GNNs) to forecast HER2 status using whole-slide images of routinely stained slides. The model was tested on the TCGA and two independent test datasets, HER2C and Nott-HER2, and achieved AUC values of 0.75 and 0.80, respectively. Su et al. [21] created a DL framework called the Breast Cancer Relapse Network (BCR-Net). The goal was to use the CNN DL approach on adjacent pairs of H&E and Ki67 breast cancer resection tissues for 50 patients. The results showed an impressive 80% accuracy, 79.2% F-Value, and 0.811 AUC. Liu et al. [22] developed and validated ML models to predict changes in visual acuity and keratometry two years after corneal crosslinking (CXL) for progressive keratoconus. Using patient demographics, visual acuity, spherical equivalence, and Pentacam parameters from 277 eyes in the Collected dataset from Aier Eye Hospital of Wuhan University (AEHWU, Wuhan, Hubei province, China), the study found that the XGBoost model produced the most accurate predictions for corrected distance visual acuity (CDVA) and maximum keratometry (Kmax) changes, with R-squared values of 0.9993 and 0.9888 for the testing set and 0.8956 and 0.8382 for the validation set. This approach significantly improved prediction accuracy by incorporating key baseline metrics such as CDVA and keratometry ratios. Botlagunta et al. [23] used ML techniques such as LR, KNN, DT, RF, SVM, GB, and XGBoost to classify and diagnose breast cancer metastasis on clinical data. They achieved 83% accuracy, 83% precision, 100% recall, an 85% F-Value, and 0.87 AUC on medical Metastatic Breast Cancer (MBC) data. Dammu et al. [24] utilized DL techniques, specifically CNN, to predict pathological complete response, residual cancer burden, and progression-free survival in breast cancer patients. The model achieved 81% accuracy, 68% sensitivity, 97% specificity, a 76% F-Value, and 0.83 AUC on the I-SPY-1 TRIAL dataset. Table 1 provides a summary of the examined state-of-the-art works.

### 1.2. Research Gap and Motivation

Relapse prediction using histopathological images and ML/ensemble learning (EL) is very important in the setting of HER2-positive breast cancer diagnosis. By applying ML algorithms to these pictures, important features may be extracted, enabling the detection of subtle patterns and traits linked to the development and return of diseases. By analyzing many image attributes, ML algorithms can shed light on the biological mechanisms that cause cancer to relapse [25,26,27]. EL offers a solid structure for combining many ML models to improve the precision of forecasts. When trying to predict when breast cancer may recur, EL approaches can be employed to merge many models that were trained on different datasets or using various algorithms. The predictive model’s capacity to generalize is improved, and the risk of overfitting is decreased by employing several modeling approaches. Using EL approaches, which combine the insights of many models to provide more accurate predictions, can enhance clinical decision-making in treating HER2-positive breast cancer.

This study aims to address the challenge of predicting breast cancer relapse in HER2-positive patients by employing multimodal analysis of histopathology images. Histological examinations are the gold standard for detecting breast cancer. The conventional, labor-intensive, and error-prone histopathology method relies heavily on histopathologists’ subjective expertise. Building a reliable computer-assisted system capable of autonomously analyzing histological images for cancer diagnosis and prognosis is of the utmost importance. According to the researchers, we may be able to glean insights from images of tissues that reveal the disease’s progression or return by merging deep neural networks with ML algorithms. We aim to develop prediction models that can accurately identify patients with a higher likelihood of experiencing a relapse by analyzing the TCGA database with HER2-positive diagnosis. By bringing together researchers from different fields, we hope to increase the accuracy of breast cancer management predictions and pave the way for more individualized approaches to therapy.

### 1.3. Objective and Contribution

It is critical to use basic numerical characteristics that accurately characterize histological pictures without changing their visual appearance, which might lead pathologists to make incorrect diagnoses to save computing costs. We overcame these challenges by detecting the color features of histopathological pictures using a Wavelet Multi-sub-band Co-occurrence Matrix and extracting texture information from low-order color moments. This research aims to develop a model for the early detection of breast cancer relapse using ML and DL techniques. The study aims to accurately characterize histopathological images by extracting color and texture features without altering their visual appearance, thereby minimizing the risk of misdiagnosis and reducing computational costs. Two stages of experiments are carried out in this study. In stage-1, six basic ML approaches, including SVM, LR, decision tree (DT), random forest (RF), adaptive boosting (AdaBoost), and extreme gradient boosting (XGBoost), are employed, whereas, in stage-2, these six ML approaches are ensembled with weighted averaging, soft voting, and hard voting to achieve the targeted improved predictive outcomes in terms of accuracy, precision, etc. These are our contributions to this study:

- The introduction of an ensemble ML approach for enhanced breast cancer relapse prediction of histopathological images.
- The creation of an ensemble model that reduces training time and optimizes performance.
- The consideration of various approaches for better image pre-processing and extracting more relevant features for prediction.
- An improvement in patient outcomes by assisting doctors in making better treatment decisions.

### 1.4. Paper Structure

The following is the structured analysis of this proposed study: Section 2 details the methods used in this study, including the suggested dataset. The design, flowchart, block diagrams, and working principle of the proposed work are covered in Section 3, which addresses the architectural aspect of the research. In contrast to the relevant outcomes examined in this study, Section 4 details the fruitful examination of the proposed work. Section 5 concludes the analysis by outlining a practical expansion of the suggested effort.

## 2. Materials and Methods

The research methodology and materials employed in this study are thoroughly elucidated in this section. The dataset, pre-processing procedures, feature extraction employing diverse methodologies, and the different ML and EL algorithms employed may be located in the respective subsections.

### 2.1. Dataset Description

A total of 123 individuals with HER2-positive breast cancer diagnoses were included in this research [28]. The Cancer Genome Atlas (TCGA) database was used to obtain the histology images stained with hematoxylin and eosin (H&E). It was shown that 26% of the people whose lymph nodes tested positive for breast cancer were younger than 50 years old. There is a total of 88 H&E-stained whole slide images (WSIs) preserved in the formalin-fixed paraffin-embedded (FFPE) format and 53 flash-frozen WSIs of breast cancer. There were five cases of relapse or metastasis among these WSIs. Patients with relapse or metastasis are represented by positive samples (labeled 1), whereas patients who did not have these problems are represented by negative samples (labeled 0). This makes it easier to explain and calculate what follows. To address the significant imbalance between relapse (positive) and non-relapse (negative) samples, we applied the Synthetic Minority Over-sampling Technique (SMOTE) to the training set. SMOTE generates synthetic samples for the minority class to balance the dataset, which helps in improving the model’s specificity and overall predictive performance. Table 2 provides a summary of the TCGA dataset that was evaluated.

### 2.2. Methodology

The current work utilizes the Contrast Limited Adaptive Histogram Equalization (CLAHE) method to enhance picture contrast. Subsequently, the Two-Dimensional Wavelet Packet Transform (2DWPT) integrated Multi-sub-bands Co-occurrence Matrix (WMCM) is used to extract texture characteristics from the images. Next, the collected features are evaluated using six distinct ML classifiers: SVM, LR, RF, DT, AdaBoost, and XGBoost. These classifiers are used as the base learners to make the first predictions. Three ensemble strategies, weighted averaging, hard voting, and soft voting are used to make the final prediction based on the initial predictions obtained using these ML classifiers.

#### 2.2.1. Preprocessing: CLAHE

Splitting an image into tiny, non-overlapping tiles is what contrast-limited adaptive histogram equalization (CLAHE) does to boost the local contrast [29,30]. CLAHE is used to enhance the contrast of histopathological images. This technique is chosen because it improves the local contrast and enhances the visibility of features without amplifying noise, making it suitable for medical images where subtle differences are crucial. Each tile’s pixel intensity values are used to create a histogram. In order to keep noise levels from getting too high, the cumulative distribution function of the histogram for each tile is clipped at a certain limit (CP Limit). Using clipping ensures that the brightness of each pixel stays below a certain limit. After that, we use the clipped CDF to build a lookup table that converts the raw intensity values to their histogram-equalized equivalents. Finally, the image is transformed pixel-by-pixel using the lookup table, which improves the local contrast without changing the image’s overall quality. One may control the amount of augmentation and the dimensions of the local regions by adjusting parameters like the tile size and clipping limit. Because of this quality, CLAHE is very effective at improving the aesthetic appeal of photographs with dynamic contrast and lighting. The detailed working of CLAHE is as follows:

- Divide the original image into small, non-overlapping tiles.
- For each tile, compute the cumulative distribution function (*C*) as in Equation (1):
  (1) $$C\left(i\right)=\frac{\sum_{k=0}^{i}H(k)}{M\times N}$$
  where $C\left(i\right)$ is the cumulative distribution function at intensity level *i*, H(k) is the histogram value at intensity level *k*, and M×N is the dimension of the image. $H\left(k\right)$ can be defined as Equation (2).
  (2) $$H\left(k\right)=\sum_{m=1}^{M}\sum_{n=1}^{N}\left\{\begin{array}{c} 1,\;if\;the\;pixel\;intensity\;at\;position\;\left(m,n\right)=k\\ 0,\;Otherwise \end{array}\right.$$
- Clip *C* to limit the contrast level for the current intensity level *k* as in Equation (3).
  (3) $$C_{clipped}\left(k\right)=Clip(C\left(i\right),0,{Clip}_{max})$$
  where $Clip(C\left(i\right),\;0,\;{Clip}_{max})$ is the method used to limit the cumulative distribution function ($C\left(i\right)$) in the range of [0, ${Clip}_{max}$]. ${Clip}_{max}$ is the controlling parameter for controlling the maximum contrast enhancement.
- Calculate the Histogram equalization Lookup Table (*ELT*) for the calculated $C_{clipped}\left(k\right)$ by rounding the value to the nearest integer as in Equation (4)
  (4) $$ELT\left(k\right)=Round(C_{clipped}\left(k\right)\times 255)$$
- Apply ELT to each individual pixel to find the enhanced pixel value.

CLAHE is a potent technique that effectively enhances the contrast of an image while preserving its original local characteristics. One may control the degree of enlargement and the size of the specific regions being analyzed by modifying parameters such as the tile size and clipping limit.

#### 2.2.2. Feature Extraction: 2D-WPT

The current work employs the 2D wavelet packet transformation (2D-WPT)-motivated Wavelet Multi-sub-bands Co-occurrence Matrix (WMCM) to extract the texture-based features from the images [21,31]. 2DWPT is used to decompose images into multiple sub-bands, allowing for capturing both spatial and frequency information. This method is selected because it effectively captures texture variations at different scales, which is important for histopathological images. WMCM is employed to extract texture features from the decomposed sub-bands. This approach is chosen because it captures the spatial relationships of pixel intensities, providing rich texture information that aids in accurate classification. The feature extraction procedure using the WMCM has many successive processes. The input picture is first wavelet-transformed into many sub-bands. The several sub-bands, each corresponding to a distinct frequency component, represent details in the picture. After that, the Co-occurrence Matrix (CM) is calculated for every sub-band, which records the spatial correlations between pixel intensities across different dimensions and orientations. Within each sub-band, the CM measures the frequency of various pixel value combinations. The WMCM unifies texture information across several frequency domains by combining the CMs from all sub-bands. It is possible to extract a collection of texture characteristics from the WMCM. Some of these properties may be statistical measurements that tell us much about the image’s texture, such as energy, correlation, contrast, and entropy. Feature extraction is a strong tool for image analysis and pattern recognition tasks. It uses the multi-scale and multi-directional information stored in the WMCM to capture texture features. The working of the WMCM model is shown below.

Step-1: Creating the WMCM

Initially, the 2D WPT model is executed to divide the input image into multiple 256 × 256 pixels sub-bands. The sub-bands are High–High (*HH*), High–Low (*HL*), Low–High (*LH*), and Low–Low (*LL*) bands. *HH* illustrates the sub-band with high frequency in both horizontal and vertical directions, *HL* shows the high-frequency information in the horizontal direction and low-frequency information in the vertical direction, *LH* shows the noise, *HL* represents the horizontal detailed sub-images, and *LH* represents the low- and high-frequency information in the horizontal and vertical directions, respectively. Similarly, *LL* represents the low-frequency information in both directions. These four bands can be found using Equations (5)–(8).

(5) $$HH=(P*\varphi^{d}(x,y))$$

(6) $$HL=(P*\varphi^{v}(x,y))$$

(7) $$LH=(P*\varphi^{h}(x,y))$$

(8) LL=(P∗∅(x,y))

where ∗ is the convolution operation, ∅ is the scaling function, φ is the wavelet function, *x* is the scaling parameter, and y is the translation parameter. $\varphi^{v}$, $\varphi^{h}$, and $\varphi^{d}$ are wavelet functions in the vertical (*v*), horizontal (*h*), and diagonal (*d*) directions, respectively.

- Calculate the Gray-Level Co-Occurrence Matrix (*GLCM*) for each pixel (*i*,*j*) sub-bands (*SB*s) for *HH*, *HL*, *LH*, *LL* using Equation (9) with $H\left(a,b\right)$ as the intensity value at any position (*a*, *b*). *M* and *N* are the dimensions of the image.
  (9) $${GLCM}_{SB}\left(i,j\right)=\sum_{m=1}^{M}\sum_{n=1}^{N}\left\{\begin{array}{c} 1,\;if\;H\left(a,b\right)=i\;,H\left(a^{\prime },b^{\prime }\right)=j\\ 0,\;Otherwise \end{array}\right.$$
  (10) $$a^{\prime }=a-\nabla$$
  (11) $$b^{\prime }=b-\nabla$$
  where the ∇ is the distance from the initial position (*a*, *b*), and *SB* is the sub-band, which can be represented as SB∈{LL,LH,HL,HH}.
- Combine the *GLCM* values to form the *WMCM* using Equation (12) with *K* as the total number of sub-bands available for the corresponding image.
  (12) $$WMCM(i,j)=\sum_{k=1}^{K}{GLCM}_{{SB}_{k}}\left(i,j\right)$$

Step-2: WMCM-based Texture Feature Extraction Process

WMCM-based texture features can be calculated using eleven different parameters including Small Gray Level with Small and Big Detail advantage denoted as *SGSDA* and *SGBDA*, respectively, Gray-Level Average (*GLA*), Detail-Level Average (*DLA*), Gray-Level and Detail-level Mean Square Error denoted as *GLMSE* and *DLMSE*, respectively, Correlation (*C*), Energy (*E*), Entropy (*En*), Contrast (*Co*), and Homogeneity. These parameters can be calculated using Equations (13)–(25) with (*i*,*j*) as the intensity level, *H*(*i*,*j*) as the value at intensity (*i*,*j*), $\vartheta \left(i\right)\;and\;\vartheta (j)$ as the marginal distribution along rows and columns, respectively, and L as the total number of intensity levels, which can be 8, 16, or 32. In the current study, the total intensity level is 16.

(13) $$SGSDA=\sum_{i=1}^{L}\sum_{j=1}^{L}H\left(i,j\right),(i^{2}.j^{2})$$

(14) $$SGBDA=\sum_{i=1}^{L}\sum_{j=1}^{L}H\left(i,j\right).(\frac{j^{2}}{i^{2}})$$

(15) $$GLA=\sum_{i=1}^{L}i.\vartheta (i)$$

(16) $$DLA=\sum_{j=1}^{L}i.\vartheta (j)$$

(17) $$\vartheta \left(i\right)=\frac{\sum_{j=1}^{L}GLCM(i,j)}{\sum_{i=1}^{L}\sum_{j=1}^{L}GLCM(i,j)}$$

(18) $$\vartheta \left(j\right)=\frac{\sum_{i=1}^{L}GLCM(i,j)}{\sum_{i=1}^{L}\sum_{j=1}^{L}GLCM(i,j)}$$

(19) $$GLMSE=\sqrt{\frac{\sum_{j=1}^{L}{\left(i-GLA\right)}^{2}.\vartheta \left(i\right)}{\sum_{i=1}^{L}\vartheta \left(i\right)}}$$

(20) $$DLMSE=\sqrt{\frac{\sum_{j=1}^{L}{\left(i-DLA\right)}^{2}.\vartheta \left(j\right)}{\sum_{i=1}^{L}\vartheta \left(j\right)}}$$

(21) $$C=\frac{\sum_{i,j=1}^{L}i.j.WMCM\left(i,j\right)-\mu_{a}\mu_{b}}{\sigma_{a}.\sigma_{b}}$$

(22) $$Co=\sum_{i,j=1}^{L}{\left(i-j\right)}^{2}.WMCM(i,j)$$

(23) $$E=\sum_{i,j=1}^{L}{\left(WMCM\left(i,j\right)\right)}^{2}$$

(24) $$En=-\sum_{i,j=1}^{L}WMCM\left(i,j\right)\log\left(WMCM\left(i,j\right)\right)\;$$

(25) $$H=\sum_{i,j=1}^{L}\frac{1}{1+(i-j)}WMCM\left(i,j\right)$$

After calculating the parameters above, the corresponding intensity level’s texture feature (*TF*) can be calculated by normalizing the parameters. The *TF* for each intensity can be calculated using Equation (26).

(26) $${TF}_{i,j}=\omega_{1}.SGSDA+\omega_{2}.SGBDA+\omega_{3}.GLA+\omega_{4}.DLA+\omega_{5}.GLMSE+\omega_{6}.DLMSE+\omega_{7}.C+\omega_{8}.Co+\omega_{9}.E+\omega_{10}.En+\omega_{11}.H$$

(27) $$\omega_{1}+\omega_{2}+\omega_{3}+\omega_{4}+\omega_{5}+\omega_{6}+\omega_{7}+\omega_{8}+\omega_{9}+\omega_{10}+\omega_{11}=1$$

Based on the *TF* for each intensity (*i*,*j*), the final texture feature for the entire image sized *M* × *N* can be quantified as in Equation (28):

(28) $$TF=\left[\begin{array}{ccc} {TF}_{i,j} & \cdots & {TF}_{i,M}\\ \vdots & \ddots & \vdots \\ {TF}_{i,N} & \cdots & {TF}_{N,M} \end{array}\right]$$

#### 2.2.3. Employed ML Approaches

Supervised learning methods such as SVM frequently identify the optimal hyperplane to separate classes in high-dimensional space in classification challenges. A logistic function is a binary dependent variable in LR, a statistical model used to estimate the probability of a particular event occurring. DTs are representations of the chronological and logical progression of healthcare issues. RF constructs and amalgamates several DTs to enhance classification accuracy and mitigate overfitting. XGBoost is a scalable and efficient implementation of a gradient-boosting framework designed to optimize computation time and enhance predictive accuracy. AdaBoost focuses on misclassified samples to combine the outputs of weak classifiers, generating a strong classifier.

Six ML classifiers are chosen for their diverse strengths in handling different types of data and their proven effectiveness in medical image analysis: SVM, effective in high-dimensional spaces and robust against overfitting, particularly with clear margins of separation; LR, a simple yet powerful classifier for binary outcomes, known for its interpretability and effectiveness when the relationship between features and the target variable is approximately linear; RF, an ensemble method that builds multiple DTs to achieve more accurate and stable predictions, chosen for its robustness and high accuracy; DT, a non-parametric model that is easy to interpret and understand, selected for its simplicity and effectiveness in capturing non-linear relationships; AdaBoost, which combines weak classifiers to create a strong classifier, improving model accuracy by focusing on difficult-to-classify samples; and XGBoost, a powerful gradient-boosting algorithm that is efficient, scalable, and capable of capturing complex patterns. Combining these techniques ensures a robust methodology that enhances image quality, extracts meaningful features, and employs diverse classifiers to handle various aspects of the data.

#### 2.2.4. Employed EL Approaches

EL integrates the outputs of several separate models to forge a more robust forecast. The ensemble strategies are used to improve the overall predictive performance by combining the strengths of individual classifiers, thereby reducing the risk of overfitting and improving generalization to new data. Three ensemble strategies are employed to enhance predictive performance: weighted averaging, soft voting, and hard voting. Weighted averaging combines the predictions of multiple classifiers by assigning different weights to each classifier based on their performance, leveraging their strengths to produce more accurate predictions. Soft voting averages the predicted probabilities of each classifier to make the final prediction, effectively combining probabilistic outputs for better performance. Hard voting aggregates the majority class predicted by each classifier to make the final decision, chosen for its simplicity and effectiveness in scenarios where the majority rule is reliable [32,33]. The goal of weighted averaging is to integrate the predictions of several models into a single score, with the weights assigned to each model based on their performance or other criteria. The ensemble prediction $\hat{p}$ is mathematically calculated as shown in Equation (29).

(29) $$\hat{p}=\sum_{k=0}^{n}{Wt}_{k}{\hat{p}}_{k}$$

Here, *n* is the number of models, ${\hat{p}}_{k}$ is the prediction of the *k*th model, and ${Wt}_{k}$ is the weight assigned to the kth model.

In soft voting, the predictions of several models are combined by averaging their anticipated class probabilities, with the confidence level of each model being used to weight the probability estimations. When all of the models’ class probabilities are averaged, the ensemble prediction results. Predictions using hard voting are based on majority voting, with the class with the most votes from the individual models being the final forecast. The mathematical mean (or most frequent class) of all the individual model predictions is the ensemble prediction. Conventional ML techniques like DTs, LR, SVMs, etc., may all benefit from these ensemble methods. Improved predictive performance and resilience are common outcomes of EL, combining many models’ predictions by weighted averaging, soft voting, or hard voting.

## 3. Proposed Model

The suggested model for accurate cancer detection is built using a set of well-planned stages that use ML methods to boost precision. As the first step in data preparation, we remove photos with a blank ratio higher than 30%. Images with a lot of empty space are not going to be considered for categorization until this requirement is met. This phase greatly improves the accuracy of the following predictions by keeping just the most pertinent and instructive pictures. Next, the photos’ contrast is improved using the CLAHE method, which stands for Contrast-Limited Adaptive Histogram Equalization. By adjusting the intensity levels, CLAHE makes key characteristics more visible, which is essential for correct categorization and extensive texture analysis.

The feature extraction process involves capturing complex texture patterns from the improved pictures using the Wavelet Multi-Channel Method (WMCM). In order to extract texture-based elements that are crucial for differentiating between non-cancerous and malignant pictures, WMCM, a variation of the 2D Wavelet Packet Transform (2DWPT), decomposes the image into several frequency sub-bands. SVM, LR, DT, RF, AdaBoost, and XGBoost are six classifiers that are used throughout the model training phase. Based on the retrieved textural information, each classifier acts as a base learner, first predicting cancer status. Improving prediction accuracy and resolving any discrepancies among these fundamental classifiers are achieved through the use of ensemble machine learning techniques. One of these methods is weighted averaging, which takes into account the relative performance of each base classifier and uses that information to combine their predictions. Another is hard voting, which uses the aggregated class labels to choose the most frequently predicted class, and the third is soft voting, which uses the average of the probability estimates to obtain the final prediction.

**Figure 1:**
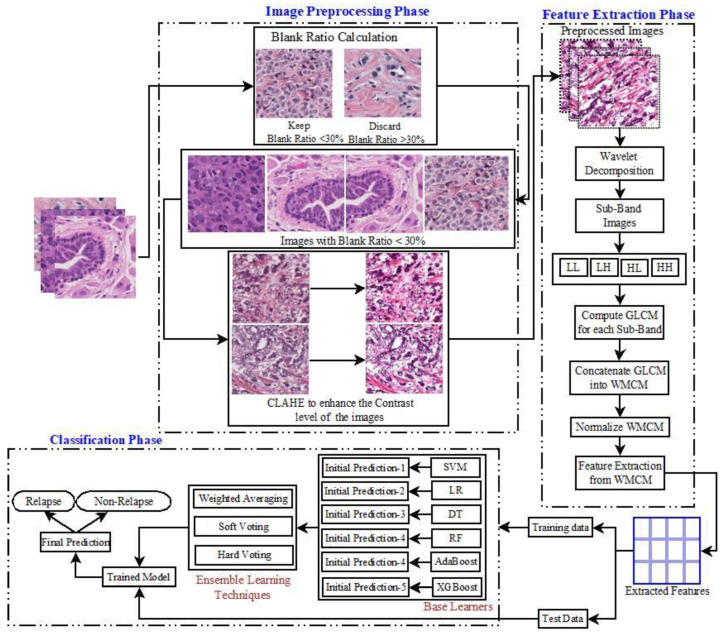
Block diagram of the proposed model.

The balanced dataset is split into two halves, the training set and the test set, according to an 80:20 ratio. About 102 samples do not experience relapse, whereas 101 samples do (using synthetic samples produced by SMOTE), for a total of 203 samples in the training set. The test set includes 51 samples, out of which 26 were from relapses (including manufactured samples) and 25 were from non-relapses. A complete assessment of the model’s efficacy may be achieved thanks to its balanced distribution, guaranteeing that both sets have a representative mix of classes. Accuracy, precision, recall, and F-Value are some of the measures used to evaluate the model’s performance in properly classifying relapse and non-relapse instances. A comprehensive evaluation follows the suggested model’s robust training using various classifiers and ensemble approaches, which begins with a comprehensive preprocessing phase. The model next undergoes sophisticated feature extraction. Figure 1 shows the proposed model’s workflow, and Algorithm A1 (in Appendix A) provides the pseudocode for the operations’ sequence.

## 4. Empirical Analysis

Several assumptions are incorporated into the evaluation of this proposed ensemble ML method. A new ensemble ML method was developed to improve the evaluation metrics by applying six traditional ML techniques to the data generated via wavelet decomposition. The proposed idea was developed using two different computer systems. The main computer, System-1(S1), is a desktop PC with various specs, including an Intel Core i7 CPU, 32 GB of RAM, 1 TB of solid-state drive storage, and Ubuntu 20.04. The secondary system, System-2 (S2), has an Intel Core i5 CPU, 8 GB of RAM, 256 GB of primary SSD, 512 GB of secondary SSD, and runs Windows 10. A thorough empirical analysis of the collected data should be conducted as part of any endeavor. Using a systematic experimental approach, these metrics aim to construct a class confusion matrix that compares the actual results to predicted ones. The confusion matrix uses the letters *T_A_* and *T_B_* to represent true positives and negatives and *F_A_* and *F_B_* to represent false positives and false negatives. The study’s performance indicators for categorization include *MCC*, *F-Value*, *Accuracy*, *Precision*, *Sensitivity*, and *Specificity*. Equations (30)–(35) detail these metrics’ definitions [34,35].

(30) $$Accuracy=\frac{T_{A}+T_{B}}{T_{A}+T_{B}+F_{A}+F_{B}}$$

(31) $$Precision=\frac{T_{A}}{T_{A}+F_{A}}$$

(32) $$Sensitivity=\frac{T_{A}}{T_{A}+F_{B}}$$

(33) $$Specificity=\frac{T_{B}}{T_{B}+F_{A}}$$

(34) $$F\text{-}Value=\frac{{2\times T}_{A}}{{2\times T}_{A}+F_{B}+F_{B}}$$

(35) $$MCC=\frac{\left(T_{A}+T_{B}\right)-\left(F_{A}+F_{B}\right)}{\sqrt{\left(T_{A}+F_{A}\right)\left(T_{A}+F_{B}\right)\left(T_{B}+F_{A}\right)\left(T_{B}+F_{B}\right)}}$$

**Figure 2:**
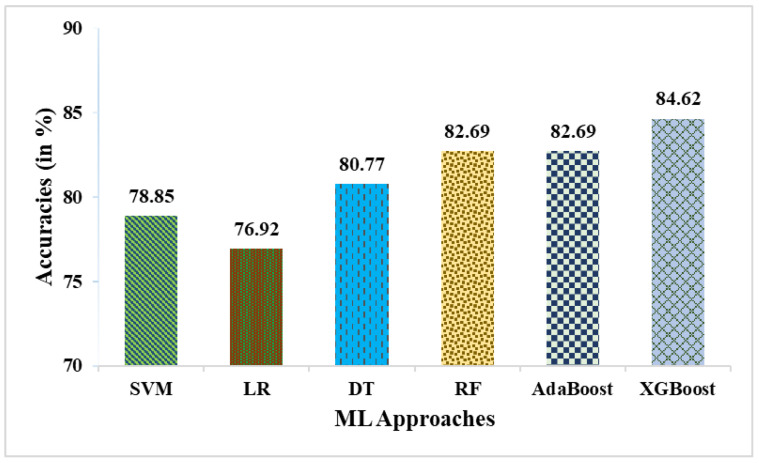
Accuracies obtained in % employing various ML approaches.

**Figure 3:**
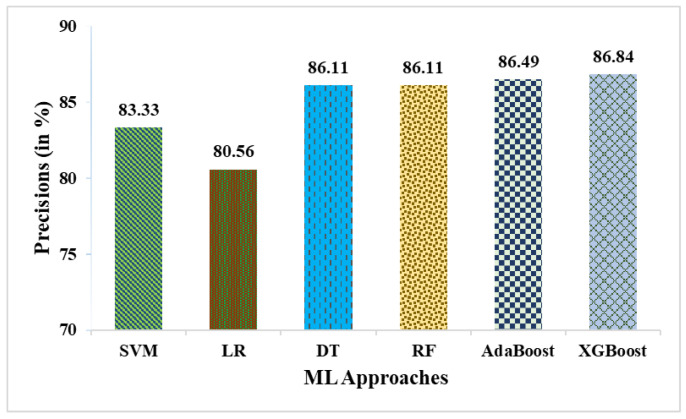
Precisions obtained in % employing various ML approaches.

**Figure 4:**
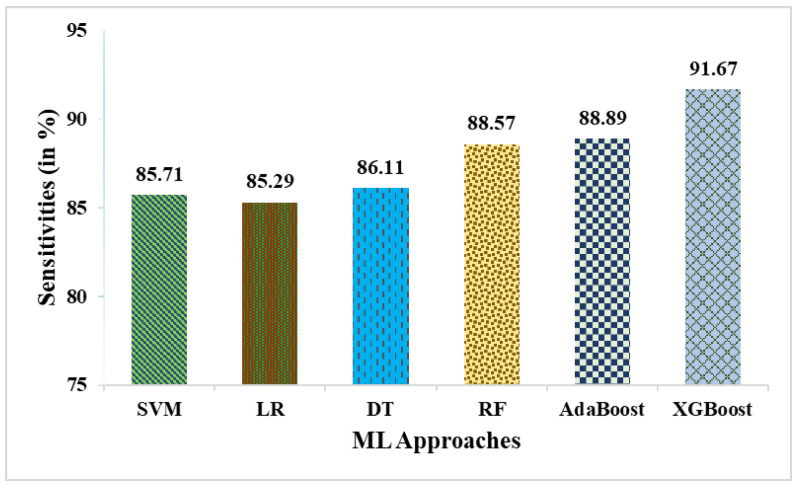
Sensitivities obtained in % employing various ML approaches.

**Figure 5:**
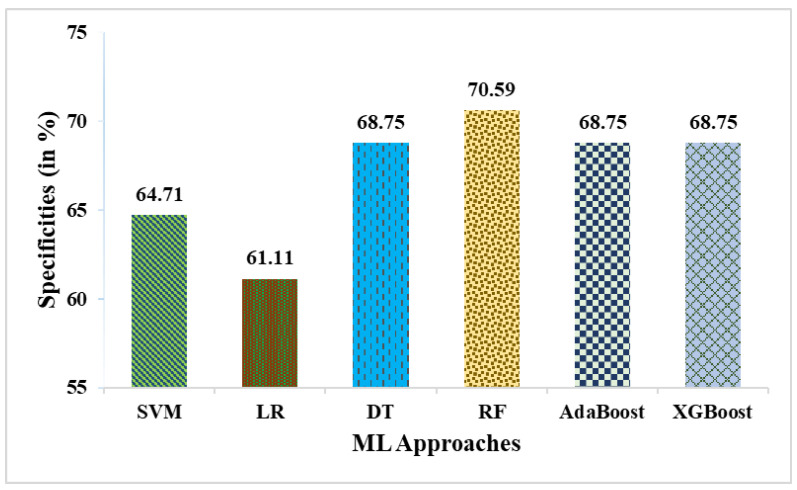
Specificities obtained in % employing various ML approaches.

**Figure 6:**
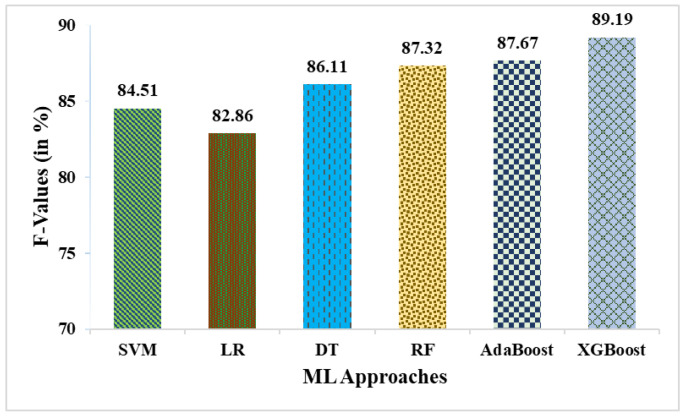
F-Values obtained in % employing various ML approaches.

**Figure 7:**
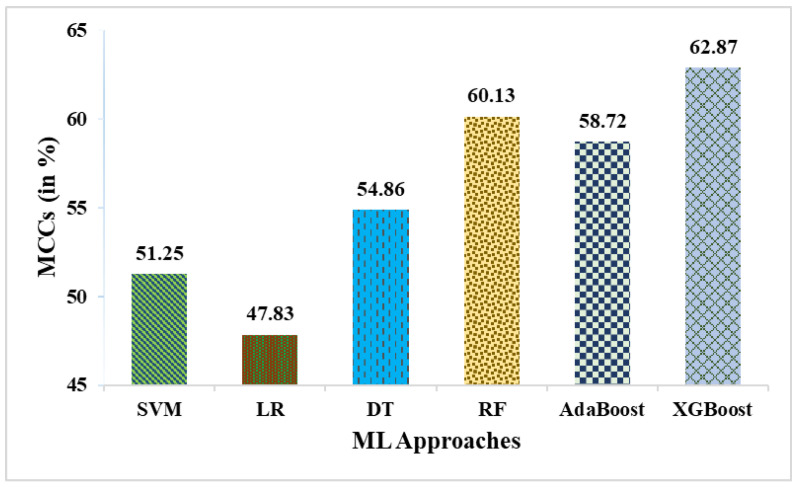
MCCs obtained in % employing various ML approaches.

This research used a two-stage experimental design. Stage-1 uses the following six ML methods: SVM, LR, DT, RF, AdaBoost, and XGBoost. In stage-2, these six ML methods are combined with weighted averaging, soft voting, and hard voting to create a more effective breast cancer relapse dataset model. Figure 2, Figure 3, Figure 4, Figure 5, Figure 6 and Figure 7 and Table 3 display the outcomes. The XGBoost method achieves an accuracy of 84.62% at its peak, as seen in Figure 2. In Figure 3, we can see the precision values that different ML methods achieved; XGBoost achieved the greatest, at 86.84%. Figure 4 shows the sensitivity values of several ML methods, with XGBoost achieving the highest value of 91.67%. The specificity values produced by different ML techniques are shown in Figure 5, with RF achieving the best value of 70.59%. Figure 6 shows the F-Values produced by several ML methods, with XGBoost achieving the best at 89.19%. Several ML methods are shown in Figure 7, with XGBoost producing the highest *MCC* value of 62.87%.

**Figure 8:**
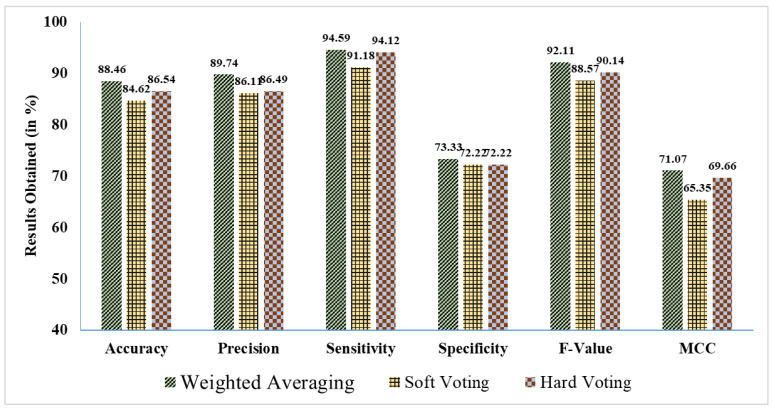
Findings acquired in % using various EL methods.

Then, in stage-2, we used weighted averaging, soft voting, and hard voting—three EL techniques—to improve the predictive outcomes. Table 4 and Figure 8 show the percentage of findings that were achieved. Compared to other ensemble ML techniques, ML approaches ensembled using weighted averaging achieve superior results (88.46% *accuracy*, 89.74% *precision*, 94.59% *sensitivity*, 73.33% *specificity*, a 92.11% F-Value, and 71.7% *MCC*). A phrase was coined to describe these improved results: the suggested ensemble ML strategy is based on the weighted average of several ML methods.

**Figure 9:**
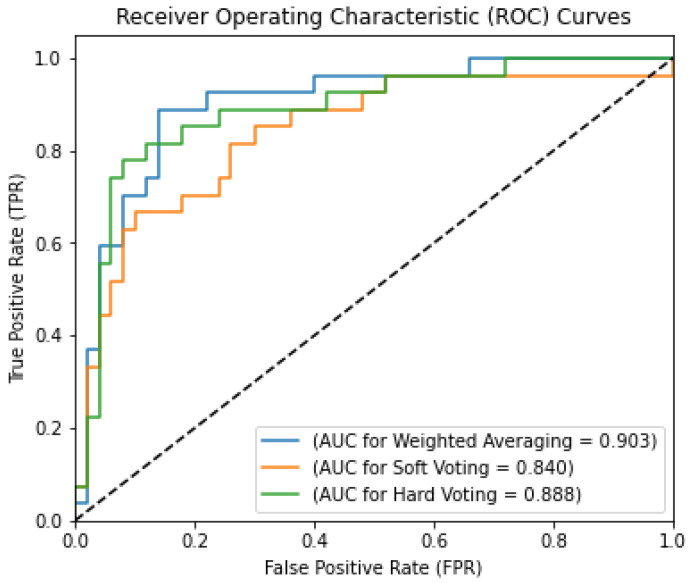
AUC values with ROC curves for the ensemble ML approaches.

Additionally, using a receiver operating characteristics (ROC) curve plot, we have verified that the basic classifiers using weighted averaging as the EL method are part of the ensemble ML strategy we suggested. A ROC curve shows how well a classification model does on every level [23]. Two measures, TPR and FPR, are shown on the graph. There is a ROC curve that shows the level of categorical uncertainty. As a result of lowering the classification criteria, the number of false and true positives rises. The AUC or area under the ROC curve indicates general efficacy. A model’s AUC indicates its propensity to assign a higher score to positive examples than negative ones. The ROC curve remains within the (0, 0) to (1, 1) range. The ROC curve and AUC value for this proposed hybrid method are shown in Figure 9. Figure 9 shows that the suggested ensemble ML method uses the ROC curve to achieve the best AUC of 0.903 out of the three EL techniques.

### 4.1. Critical Analysis

**Figure 10:**
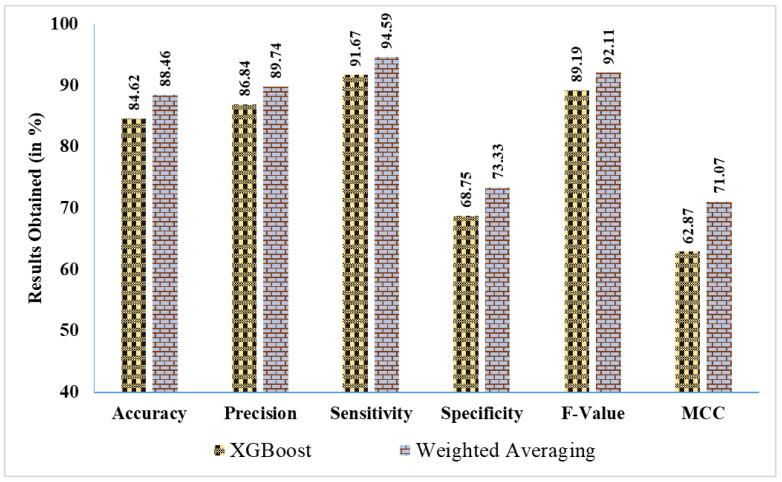
Percentage comparison of findings: best EL approach vs. best ML approach.

The proposed ensemble approaches are compared in contrast to the state-of-the-art ML classifiers to show the efficacy of the proposed work. From Table 3 and Table 4, the improvement of the proposed work can be clearly observed:

- While comparing the proposed weighted averaging of ML approaches in contrast to the ML classifiers, it outperforms the SVM, LR, DT, RF, AdaBoost, and XGBoost in terms of accuracies by ~12.19%, ~15%, ~9.52%, ~6.98%, ~6.98%, and ~4.54%, respectively.
- In the case of soft voting, it outperforms the considered ML classifiers, including SVM, LR, DT, RF, AdaBoost, and XGBoost, in terms of accuracies by ~7.32%, ~10.01%, ~4.77%, ~2.33%, ~2.33%, and ~0%, respectively.
- Comparing the hard voting ensemble technique with the ML classifiers, it outperforms SVM, LR, DT, RF, AdaBoost, and XGBoost in terms of accuracies by ~9.75 ~12.51%, ~7.14%, ~4.66%, ~4.66%, and ~2.27%, respectively.
- From the above-stated comparison, it can be clearly observed that all ensemble techniques outperform the ML classifies more or less. However, three ensemble techniques were compared to find the best-fit ensemble technique for the current work. From the results obtained, it can be concluded that the weighted averaging outperforms soft voting and hard voting techniques by ~4.54% and ~2.22%, respectively, which states that the reported weighted averaging is the best-fitting ensemble technique for the current work.
- Figure 10 presents a percentage-based comparison of results, illustrating the performance differences between the best EL approach and the best ML approach across various evaluation metrics:
  ⚬ Weighted averaging achieves an accuracy of 88.46%, outperforming XGBoost, which obtained 84.62%. This highlights a notable improvement of ~3.84%.
  ⚬ The precision for weighted averaging is 89.74%, compared to 86.84% for XGBoost, indicating an increase of ~2.9% in precision.
  ⚬ In terms of sensitivity, weighted averaging reaches 94.59%, surpassing XGBoost’s sensitivity of 91.67% by ~2.92%. This suggests that weighted averaging is better at correctly identifying positive cases.
  ⚬ Weighted averaging achieves 73.33% specificity, which is significantly higher than XGBoost’s 68.75%, resulting in an improvement of ~4.58% in identifying negative cases.
  ⚬ The F-Value for weighted averaging is 92.11%, compared to 89.19% for XGBoost, showing an enhancement of ~2.92%.
  ⚬ For *MCC*, weighted averaging scores 71.07%, significantly better than XGBoost’s 62.87%, demonstrating an improvement of ~8.2%, which reflects the overall quality of binary classifications.

### 4.2. Comparative Analysis

In addition to the above comparison among the ensemble techniques and ML classifier, we have also compared the results of using the proposed model with the weighted averaging technique with some of the existing literature, regardless of the dataset used for the work, as shown in Table 5, to support the novelty of the proposed work. When comparing the proposed work to existing literature, it becomes evident that in most situations, the proposed work outperforms the latter using all of the evaluated factors. As shown in Table 5, when comparing the proposed method with the works from the references, our proposed model outperforms most of them, particularly when using the TCGA dataset, as demonstrated in references [18,19,20]. This consistent improvement across multiple performance parameters, including *AUC*, clearly shows the effectiveness and novelty of our approach.

It could be noted that not all referenced studies provided comparable data for all metrics, such as *MCC* or *specificity*. However, we included these studies in the comparison table to provide a comprehensive view of how our model compares with various approaches used in breast cancer diagnosis research. This allows for a broader understanding of the performance of our method across different contexts and datasets. While direct comparisons were made for studies using the same dataset (TCGA), we also sought to provide context by including studies that used different datasets but evaluated similar performance parameters. This further reinforces the robustness and adaptability of our proposed method.

## 5. Conclusions

It is essential to accurately forecast the likelihood of relapse and metastasis in each patient to choose the most effective adjuvant treatment and initiate early intervention strategies since the relapse of breast cancer is directly associated with death. Patients who have had surgery for breast cancer are attracting more and more attention from researchers interested in computer-assisted diagnosis and prognosis prediction using H&E pathology images. To assess the possibility of breast cancer metastasis and relapse using histopathological images, we developed an innovative structure in this study that uses EL, ML, and image features. It is essential to employ fundamental numerical features that properly characterize histological images without altering their visual appearance to avoid misleading pathologists into making erroneous diagnoses to cut computer expenses. We overcame these obstacles by extracting texture information from low-order color moments and utilizing a Wavelet Multi-sub-band Co-occurrence Matrix to detect the color characteristics of histological images. We selected the most effective prediction models and extracted the most valuable characteristics using cross-validation. This research used a two-stage experimental design. SVM, LR, DT, RF, AdaBoost, and XGBoost are the six ML techniques used in stage-1. In stage-2, these techniques are combined with weighted averaging, soft voting, and hard voting to produce better results. Using the TCGA data warehouse, we were able to compile information on 123 breast cancer patients who had HER2-positive results. All of these patients’ relapse and metastasis statuses were known, and they had easy access to their H&E images. After two rounds of testing, the improved outcomes of an AUC of 0.903, an F-Value of 92.11%, an MCC of 71.07%, a sensitivity of 94.59%, a specificity of 73.33%, and an accuracy of 88.46% are all achieved by the suggested ensembled ML technique.

The advantages and disadvantages of each study are inevitable. The following are the limitations of the study. The findings may not be generalizable to larger groups because of the study’s restricted sample size (123 HER2-positive breast cancer patients). Previous research has examined the impact of sample size on the dependability of machine learning models. Although SMOTE addressed the class imbalance between relapse and non-relapse samples, it is crucial to acknowledge that synthetic data may introduce noise or inadequately capture the complexities of real-world data, thus affecting model performance. The histopathological images utilized in the investigation were derived solely from a single dataset (TCGA), perhaps failing to encompass the full spectrum of breast cancer manifestations across diverse populations or contexts. Research has shown that external validation across several datasets is crucial for validating prediction models. Although ensemble learning models yield superior predictions, they often lack the interpretability of simpler models, complicating their application in clinical decision-making.

Furthermore, with the use of H&E images, clinical data, and sophisticated DL algorithms, patients with HER2-positive breast cancer may have their chances of relapse and metastasis assessed. For example, our multimodal technique might be expanded in future work to include data from histopathological imaging and gene expression analysis. We aim to employ larger cohorts from more locations to improve our training data and achieve greater generalization. This will help us validate our models on a larger scale. Future work will also validate the model’s performance on external datasets and real-world clinical samples to ensure its robustness and applicability in diverse settings.

## Figures and Tables

**Table 1:** Summary of the considered state-of-the-art works.

Reference	Methodologies	Dataset(s)	Outcomes
Sakri et al. [9]	REPTree, NB, and KNN-IBK with PSO	WPBC	Accuracy: 81.3%, Precision: 88.3%, Recall: 93.4%, F-Score: 87.7%, AUC: 0.820
Alom et al. [10]	Inception-v4, ResNet, and RCNN	BreakHis and BCD	Accuracy: 100%, Sensitivity: 100%, Specificity: 100%, AUC: 1.0
Hong et al. [11]	LR and Gaussian mixture	TCGA_TNBC, GEOD-40525, GSE40049 and GSE19783	AUC (GSE40049): 0.89, AUC (GSE19783): 0.90
Yan et al. [12]	LR	BCC at HMUCH	AUC (HER2-Positive): 0.820, AUC (TNBC): 0.785
Mosayebi et al. [13]	RF, LVQ, NB, C5.0 DT, MLP, KPCA-SVM, and SVM	MHME-ICRC	Accuracy: 81.9%, Sensitivity: 86.9%, Specificity:77.7%, F-Value: 81.6%, AUC: 0.774
Comes et al. [14]	CNN and SVM	I-SPY1 TRIAL and BREAST-MRI-NACT-Pilot	Accuracy: 85.2%, Sensitivity: 84.6%, AUC: 0.83
Sanyal et al. [15]	LSTM and XGBoost	Manually and NLP-curated	Sensitivity: 89.0%, Specificity: 84.0%, AUC: 0.94
Conde-Sousa et al. [16]	DL	HEROHE	Precision: 79%, Recall: 100%, F-Value: 79%, AUC: 0.88
Rabinovici-Cohen et al. [17]	CNN	Real-World Retrospective Dataset	Specificity: 57%, F-Value: 56%, Balanced Accuracy: 72%, PPV: 41%, NPV: 96%, Sensitivity: 93%, AUC: 0.75
Liu et al. [18]	RF, LR, and XGBoost	Clinical dataset	Accuracy: 80%, Precision: 40%, Recall: 75%, F-Value: 50%, AUC: 0.75
		TCGA	Accuracy: 73%, Precision: 33%, Recall: 60%, F-Value: 42%, AUC: 0.72
Yang et al. [19]	CNN and ResNet50	CAMS	AUC: 0.76
		TCGA	AUC: 0.72
Lu et al. [20]	GNN-based Slide Graph	TCGA	AUC: 0.75
		HER2C and Nott-HER2	AUC: 0.80
Su et al. [21]	CNN	H&E and Ki67	Accuracy: 80%, F-Value: 79.2%, AUC: 0.811
Liu et al. [22]	XGBoost	AEHWU	R-Square (CVDA): 0.9993, R-Square (Kmax): 0.9888
Botlagunta et al. [23]	LR, KNN, DT, RF, SVM, GB, and XGBoost	Medical data on MBC	Accuracy: 83%, Precision: 83%, Recall: 100%, F-Value: 85%, AUC: 0.87
Dammu et al. [24]	CNN	I-SPY-1 TRIAL	Accuracy: 81%, Sensitivity: 68%, Specificity: 97%, F-Value: 76%, AUC: 0.83

**Table 2:** An overview of the considered TCGA dataset.

Dataset	Parameters													
	Stages of Tumor			ER		PR		Lymph Node Status		Age (Years)		Outcome		Total
	I	II	III	ER−	ER+	PR−	PR+	LMN−	LMN+	<50	≥50	R	NR	
TCGA	14	77	32	33	90	50	73	58	65	34	89	5	118	123

**Table 3:** Results obtained by employing various ML approaches.

ML Approaches	Results Obtained (in %)					
	*Accuracy*	*Precision*	*Sensitivity*	*Specificity*	*F-Value*	*MCC*
SVM	78.85	83.33	85.71	64.71	84.51	51.25
LR	76.92	80.56	85.29	61.11	82.86	47.83
DT	80.77	86.11	86.11	68.75	86.11	54.86
RF	82.69	86.11	88.57	70.59	87.32	60.13
AdaBoost	82.69	86.49	88.89	68.75	87.67	58.72
XGBoost	84.62	86.84	91.67	68.75	89.19	62.87

**Table 4:** Results obtained in % by employing various EL approaches.

EL Approaches	Results Obtained (in %)					
	*Accuracy*	*Precision*	*Sensitivity*	*Specificity*	*F-Value*	*MCC*
Weighted Averaging	88.46	89.74	94.59	73.33	92.11	71.07
Soft Voting	84.62	86.11	91.18	72.22	88.57	65.35
Hard Voting	86.54	86.49	94.12	72.22	90.14	69.66

**Table 5:** Comparison of the proposed work with some state-of-the-art works.

Ref.	Dataset(s)	Comparison Parameters						
		*Accuracy* (%)	*Precision* (%)	*Sensitivity* (%)	*Specificity* (%)	*F-Value* (%)	*MCC* (%)	*AUC*
[9]	WPBC	81.3	88.3	93.4	-	87.7	-	0.820
[10]	BreakHis and BCD	100	-	100	100	-	-	1.0
[11]	GSE40049	-	-	-	-	-	-	0.89
	GSE19783	-	-	-	-	-	-	0.90
[12]	BCC at HMUCH	-	-	-	-	-	-	0.820
[13]	MHME-ICRC	81.9	-	86.9	77.7	81.6	-	0.774
[14]	I-SPY1 TRIAL, BREAST-MRI-NACT-Pilot	85.2	-	84.6	-	-	-	0.83
[15]	Manually and NLP-curated	-	-	89.0	84.0	-	-	0.94
[16]	HEROHE	-	79	100	-	79	-	0.88
[17]	Real-World Retrospective Dataset	-	-	93	57	56	-	0.75
[18]	Clinical dataset	80	40	75	-	50	-	0.75
	TCGA	73	33	60	-	42	-	0.72
[19]	CAMS	-	-	-	-	-	-	0.76
	TCGA	-	-	-	-	-	-	0.72
[20]	TCGA	-	-	-	-	-	-	0.75
	HER2C and Nott-HER2	-	-	-	-	-	-	0.80
[21]	H&E and Ki67	80	-	-	-	79.2	-	0.811
[22]	AEHWU	-	-	-	-	-	-	-
[23]	Medical data on MBC	83	83	100	-	85	-	0.87
[24]	I-SPY-1 TRIAL	81	-	68	97	76	-	0.83
[Proposed]	TCGA	88.46	89.74	94.59	73.33	92.11	71.07	0.903

## Data Availability

Publicly available datasets were analyzed in this study. These data can be found here: The dataset included for this study can be found in The Cancer Genome Atlas (TCGA) [Link: https://portal.gdc.cancer.gov/ (accessed on 22 July 2023)].

## Appendix A

**Algorithm A1:** Pseudocode of the Proposed Model Working Principle.
**Input:** Input image set (*D*), total pixel (*TP*), Gray Level intensity (8/16/24), number of images (*I*) **Output:** Cancer Relapse, Cancer Non-Relapse Procedure: Initiate the image preprocessing phase-1 to calculate the blank ratio of an image from the dataset. For *t* 1←*I* Find the number of Blank Pixels (*B*) Find the *TP* Find the Blank Ratio (*BR*) as $\frac{B}{TP}\times 100\%$ If (*BR* > 30%) Discard the image Else Keep the image EndIf Return *D’* with a set of images having *BR* < 30% EndFor Initiate the preprocessing phase-2 (CLAHE) to *D’* for enhancing the intensity level contrast Divide the original image into small, non-overlapping tiles (*ST*) For *t* 1←*ST* Calculate the Cumulative distribution function (*C*) using Equation (1). Clip the *C* to limit (${Clip}_{max}$) the contrast level for the current intensity Return image *i* EndFor Form update the dataset *D’* with enhanced Contrast level Apply *WMCM* to *D*’. For *t* ← 1 to *I* Divide the image into sub-bands SB∈{LL,LH,HL, HH} For *k* ← 1 to |*SB*| Find GLCM for each intensity (*i*,*j*) of the image ${GLCM}_{SB}\left(i,j\right)=\sum_{m=1}^{M}\sum_{n=1}^{N}\left\{\begin{array}{c} 1,\;if\;H\left(a,b\right)=i\;,H\left(a^{\prime },b^{\prime }\right)=j\\ 0,\;Otherwise \end{array}\right.$ EndFor $WMCM(i,j)=\sum_{k=1}^{K}{GLCM}_{{SB}_{k}}\left(i,j\right)$ EndFor Initiate Texture feature extraction from *WMCM* For t ← 1 to *I* Calculate SGSDA, SGBDA, GLA, DLA, GLMSE, DLMSE, C, Co, E, En,H Calculate Texture Feature (${TF}_{i,j}$) for intensity (*i*,*j*) EndFor Find *TF* by combining calculated ${TF}_{i,j}$ Split the *TF* with test size 0.2 Apply Base Learners SVM, LR, DT, RF, AdaBoost, and XGBoost to form an initial prediction. Apply Weighted Averaging, Hard Voting, and Soft Voting to form ensemble models. Evaluate the trained ensemble model over Test Data Cancer Classification.
